# Supplementary material for: Apolipoprotein-L1 G1 variant contributes to hydrocephalus but not to atherosclerosis in apolipoprotein-E knock-out mice
Source: bioRxiv. 2024 Dec 28:2024.12.28.630625. Preprint. [Version 1] doi: 10.1101/2024.12.28.630625 (PMC11722280; doi:10.1101/2024.12.28.630625)
Supplement: Supplement 1 [file media-1.pdf]

Supplementary Material

Apolipoprotein-L1 G1 variant contributes to hydrocephalus but not to atherosclerosis in apolipoprotein-E knock-out mice

Running Title: Hydrocephalus in APOL1-G1/ApoE-KO mice

Teruhiko Yoshida[1,2], Zhi-Hong Yang[3], Shinji Ashida[4], Zu Xi Yu[3], Shashi Shrivastav[1], Krishna Vamsi Rojulpote[3], Piroz Bahar[3], David Nguyen[3], Danielle A. Springer[3], Jeeva Munasinghe[5], Matthew F. Starost[6], Victoria J. Hoffmann[6], Avi Z. Rosenberg[7], Bibi Bielekova[4], Han Wen[3], Alan T. Remaley[3], Jeffrey B. Kopp[1]

[1] National Institute of Diabetes and Digestive and Kidney Diseases, NIH, Bethesda, MD,

[2] Graduate School of Medicine, The University of Tokyo, Tokyo, JAPAN,

[3] National Heart, Lung, and Blood Institute, NIH, Bethesda, MD,

[4] National Institute of Allergy and Infectious Diseases, NIH, Bethesda, MD,

[5] National Institute of Neurological Disorders and Stroke, NIH, Bethesda, MD,

[6] Office of the Director, NIH, Bethesda, MD

[7] Department of Pathology, Johns Hopkins Medical Institutions, Baltimore, MD

Corresponding author and lead contact: Teruhiko Yoshida, MD PhD;

address: 7-3-1 Hongo, Bunkyo-ku, Tokyo, JAPAN 113-8655

phone: 301-496-2988; *email:* yoshidateruhiko@g.ecc.u-tokyo.ac.jp

Table of Contents

Supplemental Tables

Supplemental Table 1. Metrics summary of single-nuclear RNA-seq

Supplemental Table 2. Concept identifiers of hydrocephalus used in All of Us analysis

Supplemental Table 3. Concept identifiers of atherosclerosis used in All of Us analysis

Supplemental Table 4. Key resource tables

Supplemental Figures

Supplemental Figure 1. Echocardiography images.

Supplemental Figure 2. Downstream factors of RICTOR showing differential expression and predicted activation of RICTOR from single-nuclear RNA-seq data from choroid plexus epithelial cells, comparing APOL1-G1 mice and WT mice.

Supplemental Figure 3. CSF cytokine panel results

Supplemental Figure 4. Weight and albuminuria levels of BAC/APOL1xApoE-KO mice among APOL1 genotypes

Supplemental Figure 5. Hydrocephalus prevalence among APOL1 genotypes in the All of Us population cohort.

Supplemental Figure 6. Atherosclerosis prevalence among APOL1 genotypes in the All of Us population cohort.

Supplemental Figure 7. BAC/APOL1-G1 genome insertion site and neighboring gene expressions in single-nucleus RNA-seq data.

Supplemental Table 1. Metrics summary of single-nuclear RNA-seq

Sample	*Estimated Number of Cells*	*Mean Reads per Cell*	*Median Genes per Cell*	*Number of Reads*	*Valid Barcodes*	*Sequencing Saturation*	*Q30 Bases in Barcode*	*Q30 Bases in RNA Read*	*Q30 Bases in UMI*	*Reads Mapped to Genome*	*Reads Mapped Confidently to Genome*	*Reads Mapped Confidently to Intergenic Regions*	*Reads Mapped Confidently to Intronic Regions*	*Reads Mapped Confidently to Exonic Regions*	*Reads Mapped Confidently to Transcriptome*	*Reads Mapped Antisense to Gene*	*Fraction Reads in Cells*	*Total Genes Detected*	*Median UMI Counts per Cell*
1_WT	6,636	104,498	1,695	693,447,828	94.80%	80.20%	96.80%	94.50%	96.50%	93.50%	90.40%	6.50%	45.80%	38.20%	45.20%	38.60%	47.70%	26,544	2,977
2_G1	15,626	83,902	1,425	1,311,056,762	95.00%	84.20%	96.70%	94.20%	96.50%	94.00%	90.90%	6.70%	42.90%	41.40%	46.40%	37.70%	51.40%	26,313	2,303

Supplemental Table 2. Concept identifiers of hydrocephalus used in All of Us analysis

Concept name (Condition)	Concept identifier
Acquired hydrocephalus	4256924
Benign intracranial hypertension	312902
Communicating hydrocephalus	440700
Congenital hydrocephalus	438244
Fetal hydrocephalus	37204822
Hydrocephalus	4043738
Non-obstructive hydrocephalus	4105343
Normal pressure hydrocephalus	432899
Obstructive hydrocephalus	440385

Supplemental Table 3. Concept identifiers of atherosclerosis used in All of Us analysis

Concept name (Condition)	Concept identifier
Atherosclerosis	44825446
Atherosclerosis	1569271
Atherosclerosis of aorta	44823124
Atherosclerosis of aorta	35207841
Atherosclerosis of coronary artery bypass graft(s) without angina pectoris	45567167
Atherosclerosis of coronary artery without angina pectoris	764123
Atherosclerosis of native arteries of extremities with intermittent claudication	1569274
Atherosclerosis of native arteries of the extremities	44828992
Atherosclerosis of native arteries of the extremities	1569272
Atherosclerosis of native arteries of the extremities with intermittent claudication	44825447
Atherosclerosis of other coronary vessels without angina pectoris	1569146
Coronary arteriosclerosis	317576
Coronary atherosclerosis	44835931
Coronary atherosclerosis due to calcified coronary lesion	45567168
Coronary atherosclerosis of native coronary artery	44830080
Coronary atherosclerosis of unspecified type of vessel, native or graft	44835932
Other and unspecified atherosclerosis	1569320
Unspecified atherosclerosis	45581810
Unspecified atherosclerosis of native arteries of extremities	1569273

Supplemental Table 4. Key resource tables

REAGENT or RESOURCE	SOURCE	IDENTIFIER
**Antibodies**		
APOL1	Genentech	5.17D12, 3.7D6+3.1C1[1]
pNKCC1	Millipore-Sigma	ABS1004
pSPAK	Millipore-Sigma	07-2273
Phospho-SGK1 (Thr256)	Thermo Fisher Scientific	44-1260G
pAkt	Cell Signaling	9271
Goat anti-Rabbit IgG (H+L) Highly Cross-Adsorbed Secondary Antibody, Alexa Fluor Plus 488	Thermo Fisher Scientific	A32731TR
SGK1	Abcam	ab43606
β-actin	Santa Cruz Biotechnology	47778
**Chemicals, peptides, and recombinant proteins**		
Doxycycline hydrochloride	Millipore-Sigma	D3072
**Critical commercial assays**		
Cholesterol E kit	Fujifilm Wako Chemicals	NC9138103
ImmPRESS HRP Horse Anti- rabbit IgG Polymer Kit, Peroxidase	Vector Laboratories	MP-7401
ImmPACT DAB EqV Peroxidase (HRP) Substrate	Vector Laboratories	SK-4103
Lipofectamine 3000	Thermo Fisher Scientific	L3000001
Mouse Cytokine/Chemokine 32-Plex Discovery Assay Array	Eve technologies	MD32
Chromium Next GEM Single Cell 3' Reagent Kit, v3.1 chemistry	10xGenomics	1000128
**Deposited data**		
Raw and analyzed single-nuclear RNA-seq data	This paper	GEO: GSE252599
**Experimental models: Cell lines**		
Human choroid plexus epithelial cells	ScienCell	1310
**Experimental models: Organisms/strains**		
BAC/APOL1-G0, G1	MSD<br>Okamoto et al.[2], Ryu et al.[3], Wakashin et al.[4]	N/A
ApoE-KO (B6.129P2-Apoe^{tm1Unc}/J)	Jackson Laboratory	002052
**Recombinant DNA**		
TRE-Empty, TRE-APOL1-G0, G1, G2 plasmids	This paper	N/A
pCMV-Tet3G plasmid	Takara Bio USA	631335
**Software and algorithms**		
CellRanger	Zheng et al.[5]	https://www.10xgenomics.com/support/software/cell-ranger
SoupX	Young et al.[6]	https://github.com/constantAmateur/SoupX

DoubletFinder	McGinnis et al.[7]	https://github.com/chris-mcginnis-ucsf/DoubletFinder
Seurat	Hao et al.[8]	https://satijalab.org/seurat/
SeuratData	Hao et al.[8]	https://satijalab.org/seurat/
Ingenuity Pathway Analysis (IPA)	QIAGEN<br>Kramer et al.[9]	N/A

1. Scales SJ, Gupta N, De Mazière AM, et al. Apolipoprotein L1-Specific Antibodies Detect Endogenous APOL1 inside the Endoplasmic Reticulum and on the Plasma Membrane of Podocytes. *Journal of the American Society of Nephrology*. 2020;31(9):2044-2064. doi:10.1681/asn.2019080829
2. Okamoto K, Rausch JW, Wakashin H, et al. APOL1 risk allele RNA contributes to renal toxicity by activating protein kinase R. *Commun Biol*. 2018;1:188. doi:10.1038/s42003-018-0188-2
3. Ryu JH, Ge M, Merscher S, et al. APOL1 renal risk variants promote cholesterol accumulation in tissues and cultured macrophages from APOL1 transgenic mice. *PLoS One*. 2019;14(4):e0211559. doi:10.1371/journal.pone.0211559
4. Wakashin H, Heymann J, Roshanravan H, et al. APOL1 renal risk variants exacerbate podocyte injury by increasing inflammatory stress. *BMC Nephrol*. Aug 27 2020;21(1):371. doi:10.1186/s12882-020-01995-3
5. Zheng GX, Terry JM, Belgrader P, et al. Massively parallel digital transcriptional profiling of single cells. *Nat Commun*. Jan 16 2017;8:14049. doi:10.1038/ncomms14049
6. Young MD, Behjati S. SoupX removes ambient RNA contamination from droplet-based single-cell RNA sequencing data. *GigaScience*. 2020;9(12)doi:10.1093/gigascience/giaa151
7. McGinnis CS, Murrow LM, Gartner ZJ. DoubletFinder: Doublet Detection in Single-Cell RNA Sequencing Data Using Artificial Nearest Neighbors. *Cell Syst*. Apr 24 2019;8(4):329-337 e4. doi:10.1016/j.cels.2019.03.003
8. Hao Y, Hao S, Andersen-Nissen E, et al. Integrated analysis of multimodal single-cell data. *Cell*. Jun 24 2021;184(13):3573-3587.e29. doi:10.1016/j.cell.2021.04.048
9. Kramer A, Green J, Pollard J, Jr., Tugendreich S. Causal analysis approaches in Ingenuity Pathway Analysis. *Bioinformatics*. Feb 15 2014;30(4):523-30. doi:10.1093/bioinformatics/btt703

Supplementary Figure 1

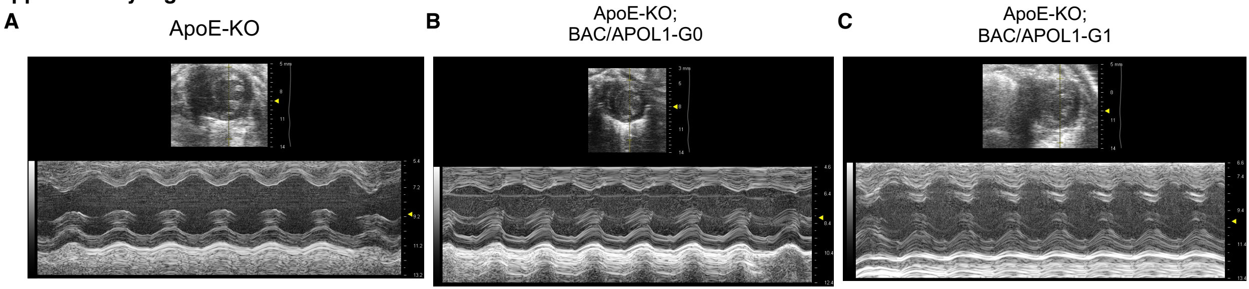

Supplemental Figure 1. Echocardiography images

(**A-C**) Representative M-mode images of echocardiography showed no difference among mice of each genotype.

Supplementary Figure 2

A

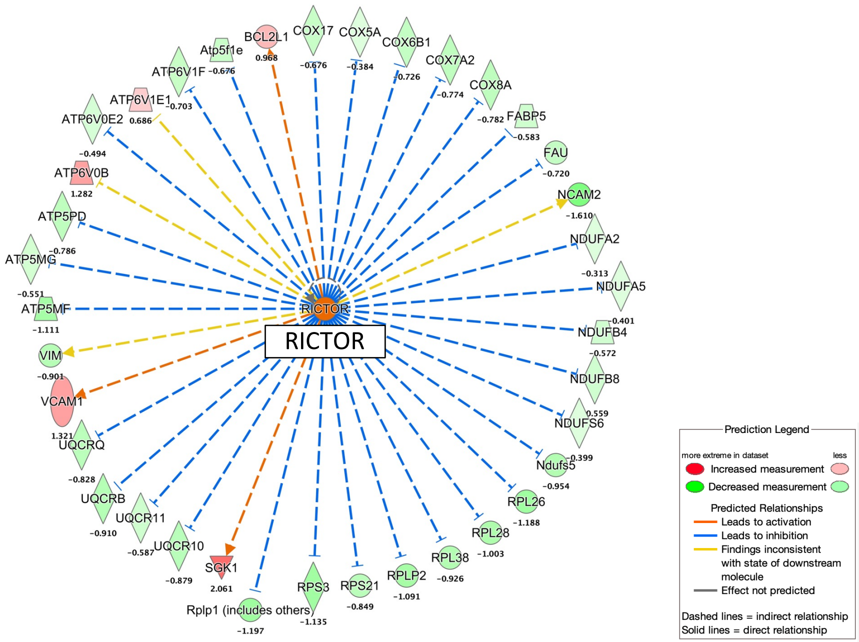

Supplemental Figure 2. Downstream factors of RICTOR showing differential expression and predicted activation of RICTOR from single-nuclear RNA-seq data from choroid plexus epithelial cells, comparing ApoE-KO; BAC/APOL1-G1 mice and ApoE-KO mice.

(**A**) Downstream factors of RICTOR indicated activation of RICTOR in choroid plexus epithelial cells of ApoE-KO; BAC/APOL1-G1 mice compared with ApoE-KO mice.

Number denotes the expression in log2 fold-change comparing ApoE-KO; BAC/APOL1-G1 mice and ApoE-KO mice.

Red denotes upregulation in ApoE-KO; BAC/ APOL1-G1, and green denotes downregulation in ApoE-KO; BAC/ APOL1-G1 mice.

Supplementary Figure 3

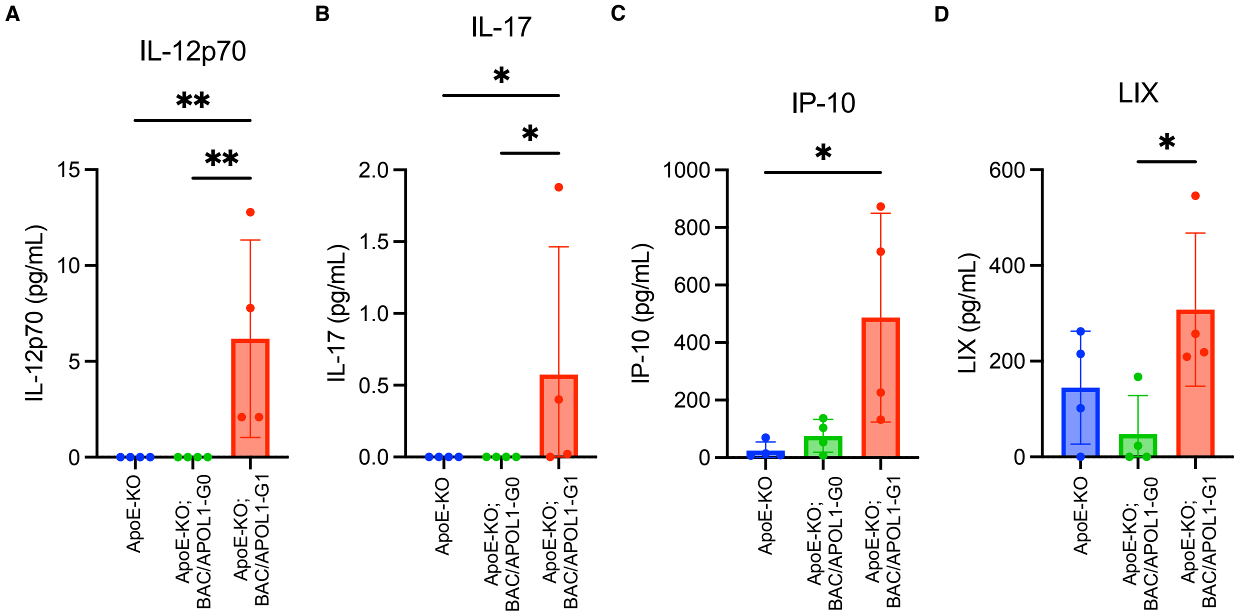

Supplemental Figure 3. CSF cytokine panel results
(**A-D**) Cytokines (IL-12p70, IL-17, IP-10 and LIX) measured in CSF showed higher levels in APOL1-G1 mice.
IP-10, Interferon gamma-induced protein 10 (CXCL10); LIX, LPS-induced CXC chemokine (CXCL5)

Supplementary Figure 4

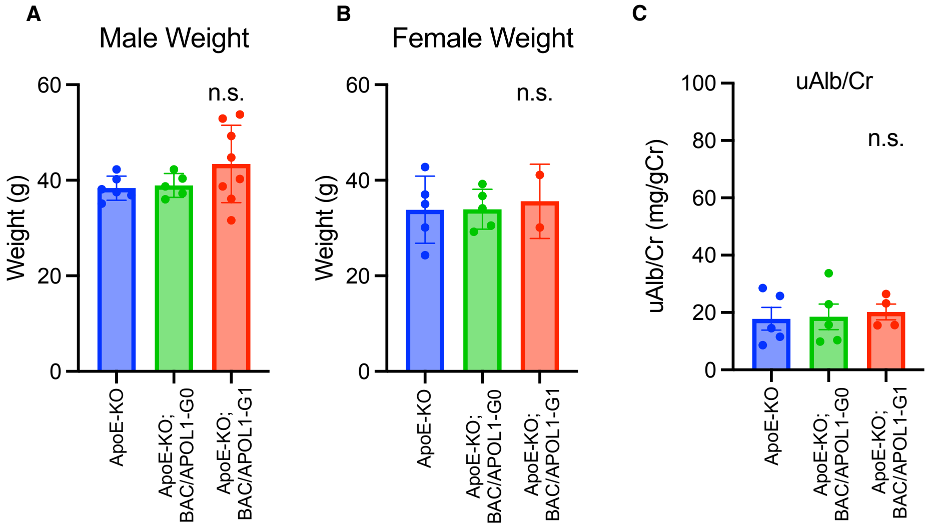

Supplemental Figure 4. Weight and albuminuria levels of BAC/APOL1xApoE-KO mice among APOL1 genotypes
(**A-B**) Weights of 9 month-old mice showed no difference among genotypes.
(**C**) Urinary albumin/creatinine ratio showed no difference among genotypes.

Supplemental Figure 5

A

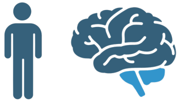

		Subjects	Case	Prevalence	Odds Ratio [95% CI]	P-value
Models	G1 allele recessive				**1.73 [1.11-2.60]**	**0.012***
	G1 allele dominant				**1.30 [1.03-1.64]**	**0.025***
	Risk allele recessive				1.19 [0.85-1.64]	0.27
	Risk allele dominant				1.19 [0.93-1.52]	0.16
APOL1 Genotype	G1/G1	2844	26	0.91%	**1.85 [1.16-2.86]**	**0.0092****
	G1 or G2/G2	4505	21	0.47%	0.94 [0.56-1.51]	0.91
	G0/G2	9594	43	0.45%	0.90 [0.62-1.30]	0.60
	G0/G1	15382	100	0.65%	1.31 [0.99-1.74]	0.057
	G0/G0	22172	110	0.50%	Ref	Ref

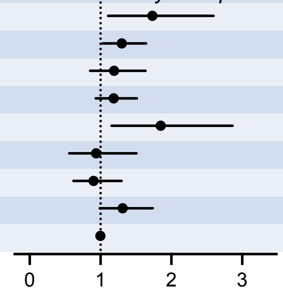

B

Univariate analysis	*All (n=54497)*	*Hydrocephalus (n=300)*	*No hydrocephalus (n=54197)*	*P-value*
Age (mean, SD)	53.0, 14.8	49.8, 14.4	53.0, 14.8	0.00021*
Male (n, %)	23292, 43%	44, 15%	23252, 43%	2.2E-16*
eGFR (mean, SD)	75.5, 30.0	63.2, 25.9	75.6, 30.0	0.0048*
G1 allele recessive (n, %)	2844, 5.2%	26, 8.7%	2818, 5.2%	0.012*
G1 allele dominant (n, %)	21590, 40%	138, 46%	21456, 40%	0.025*

C

APOL1-G1 recessive model

Multi-variate logistic regression analysis	*Coefficient*	*Odds ratio [95% CI]*	*P-value*
Age (mean, SD)	-0.030	0.97 [0.95-0.99]	0.0034*
Male (n, %)	-0.75	0.48 [0.21-0.95]	0.048*
eGFR (mean, SD)	-0.014	0.99 [0.98-0.99]	0.0032*
G1 allele recessive (n, %)	1.471	4.35 [1.93-8.88]	0.00014*

D

APOL1-G1 dominant model

Multi-variate logistic regression analysis	*Coefficient*	*Odds ratio [95% CI]*	*P-value*
Age (mean, SD)	-0.032	0.97 [0.95-0.99]	0.0017*
Male (n, %)	-0.74	0.48 [0.22-0.96]	0.0498*
eGFR (mean, SD)	-0.015	0.99 [0.98-0.99]	0.0026*
G1 allele dominant (n, %)	1.35	3.86 [2.09-7.50]	0.000028*

Supplemental Figure 5. Hydrocephalus prevalence among APOL1 genotypes in the All of Us population cohort.
(**A, B**) The prevalence of hydrocephalus stratified by APOL1 genotype and by genetic models and univariate analyses were shown. Fisher's exact test was used for APOL1 models, genotypes, and sex. t-test was used for age and eGFR.
(**C, D**) Multi-variate logistic regression analysis results for hydrocephalus odds ratios, adjusted for age, sex, and eGFR were shown with APOL1-G1 recessive model, and dominant model.

Supplemental Figure 6

A

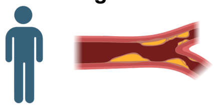

		Subjects	Case	Prevalence	Odds Ratio [95% CI]	P-value
Models	G1 allele recessive				**1.19 [1.02-1.37]**	**0.024***
	G1 allele dominant				0.98 [0.92-1.06]	0.65
	Risk allele recessive				1.04 [0.94-1.15]	0.46
	Risk allele dominant				0.99 [0.93-1.07]	0.83
APOL1 Genotype	G1/G1	2844	210	7.38%	**1.17 [1.00-1.36]**	**0.048****
	G2/G2	1141	60	5.26%	0.81 [0.61-1.06]	0.13
	G1/G2	3364	212	6.30%	0.99 [0.85-1.15]	0.88
	G0/G2	9594	631	6.58%	1.03 [0.93-1.14]	0.53
	G0/G1	15382	938	6.10%	0.95 [0.87-1.04]	0.25
	G0/G0	22172	1417	6.39%	Ref	Ref

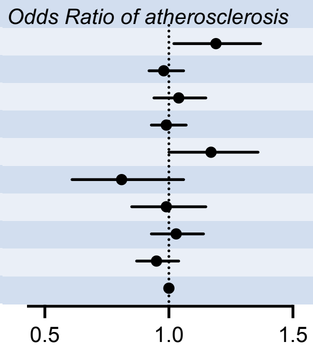

B

Univariate analysis	*All (n=54497)*	*Atherosclerosis (n=3369)*	*No atherosclerosis (n=51128)*	*P-value*
Age (mean, SD)	53.0, 14.8	65.4, 10.7	52.1, 14.6	<2.0E-16*
Male (n, %)	23292, 43%	1512, 45%	21784, 43%	0.3
eGFR (mean, SD)	75.5, 30.0	69.0, 31.1	76.9, 29.6	9.7E-9*
G1 allele recessive (n, %)	2844, 5.2%	210, 6.2%	2634, 5.2%	0.024*
G1 allele dominant (n, %)	21590, 40%	1361, 40%	20233, 40%	0.65

C

APOL1-G1 recessive model

Multi-variate logistic regression analysis	*Coefficient*	*Odds ratio [95% CI]*	*P-value*
Age (mean, SD)	0.078	1.08 [1.07-1.09]	5.5E-63*
Male (n, %)	0.40	1.5 [1.22-1.83]	8.6E-05*
eGFR (mean, SD)	-0.0072	0.993 [0.990-0.996]	2.6E-05*
G1 allele recessive (n, %)	0.21	1.23 [0.78-1.88]	0.36

D

APOL1-G1 dominant model

Multi-variate logistic regression analysis	*Coefficient*	*Odds ratio [95% CI]*	*P-value*
Age (mean, SD)	0.078	1.08 [1.07-1.09]	5.3E-63*
Male (n, %)	0.41	1.50 [1.23-1.83]	7.8E-05*
eGFR (mean, SD)	-0.0071	0.993 [0.990-0.996]	2.7E-05*
G1 allele dominant (n, %)	0.14	1.15 [0.95-1.40]	0.16

Supplemental Figure 6. Atherosclerosis prevalence among APOL1 genotypes in the All of Us population cohort.
(**A, B**) The prevalence of atherosclerosis stratified by APOL1 genotype and by genetic models and univariate analyses were shown. Fisher's exact test was used for APOL1 models, genotypes, and sex. t-test was used for age and eGFR.
(**C, D**) Multi-variate logistic regression analysis results for atherosclerosis odds ratios, adjusted for age, sex, and eGFR were shown with APOL1-G1 recessive model, and dominant model.

Supplemental Figure 7

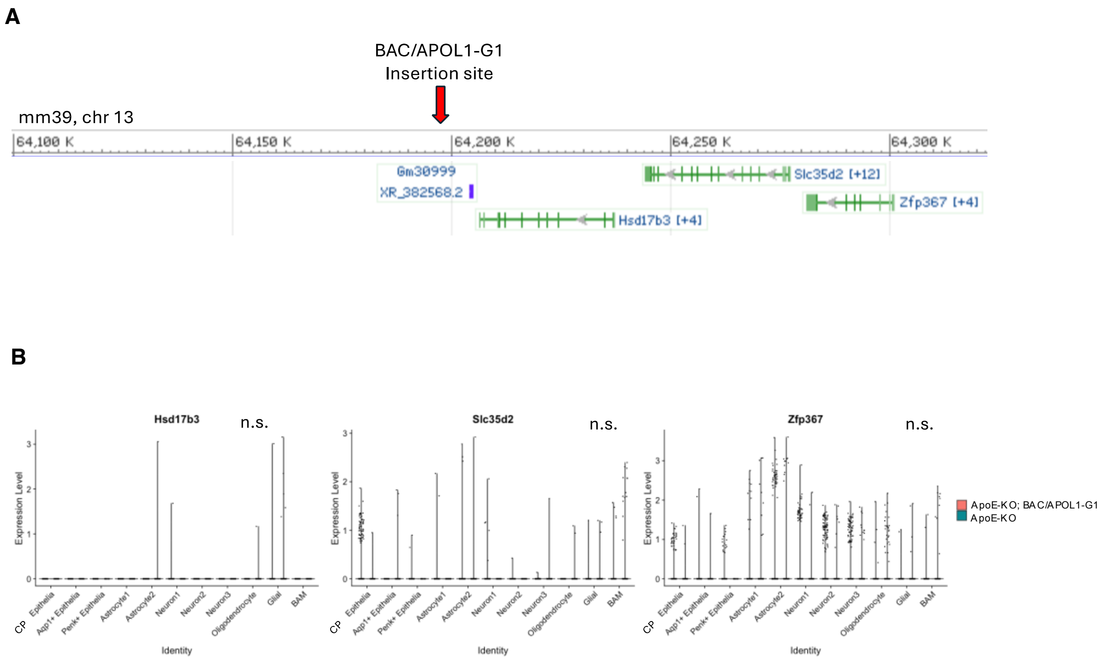

Supplemental Figure 7. BAC/APOL1-G1 genome insertion site and neighboring gene expressions in single-nucleus RNA-seq data.
(**A**) BAC/APOL1-G1 transgene insertion site was detected (mm39, chr13: 64,197,419) as shown.
(**B**) Volcano plots showed gene expression of neighboring genes (*Hsd17b3, Slc35d2, Zfp367*) around the transgene insertion site in choroid plexus epithelial cells from single-nucleus RNA-seq data.
